# Development of an AI integrated virtual reality system for reducing dental anxiety among pediatric patients

**DOI:** 10.6026/973206300212992

**Published:** 2025-09-30

**Authors:** Bhaswati Roy, Samir P.V., Aruna Kumari G., Sukabhogi Anusha, Nithya S., Anandamoy Bagchi

**Affiliations:** 1 Department of Pedodontics & Preventive Dentistry, Kalinga Institute of Dental Sciences (KIDS), Kalinga Institute of Industrial Technology (KIIT) Deemed to be University, Bhubaneswar- 751024, Odisha, India; 2 Department of Pedodontics & Preventive Dentistry, Malla Reddy Dental College for Women- Mallareddy Viswavidyapeeth, Hyderabad, India; 3 Department of Pedodontics and Preventive dentistry, Meghana Institute of Dental Sciences, Nizamabad, Telangana, India; 4 Department of Oral and Maxillofacial Pathology, ESIC Dental College and Hospital, Rohini, Delhi, India

**Keywords:** Artificial Intelligence, virtual reality, dental anxiety, pediatric dentistry, behavior management, child dental fear, interactive systems

## Abstract

Childhood dental fear prevents the successful care. This exploratory study tested an AI-based VR system in the reduction of anxiety using 60 pediatric dental patients. The MCDAS scores measured and the heartbeat rates of Group B (AI-VR) reduced significantly more than in Group A (routine care). Thus, artificial intelligence increased interaction and fun in real-time. AI-VR seems like an excellent assistant in treating dental anxiety in children.

## Background:

A specific behavioral issue that is common in dentistry among children is dental anxiety which results in a series of missed visits and poor oral health outcomes [1]. Children can be afraid because of negative interactions in the past, fear of pain and lack of familiarity with a dental environment, all of which have a negative impact on the outcome of dental procedures and the acquisition of favorable attitudes towards oral care [2]. The conventional methods like tell-show-do, distraction and positive reinforcement have demonstrated mixed efficacies and rely heavily on the skill of the dentist and the temperament of the child [3]. There has been an interest in the use of technology, particularly Virtual Reality (VR), as a non-pharmacological measure for reducing dental anxiety in children. VR offers immersive experiences that distract the child from the dental environment and make them less aware of discomfort [4]. Research has shown that VR significantly decreases behavioral distress and physiological markers of anxiety during dental procedures in children [5]. Nevertheless, traditional VR systems are not dynamic and do not adjust to the child's emotional condition in real time. The most unique application of Artificial Intelligence (AI) is its ability to adapt in real time through analysis of facial expressions, voice modulation and biosignal interpretation [6]. Combined with VR, AI enables the virtual environment to adapt according to the child's emotional and physiological reactions, thereby maximizing the distractive effect [7]. This integration offers personalization, which enhances engagement and provides greater psychological comfort for pediatric patients. The use of immersive technologies in healthcare, especially for managing procedural anxiety in children, has gained increasing attention in recent years. VR has also been studied extensively in other medical fields such as phlebotomy, vaccination and burn wound care, demonstrating reductions in anxiety and improved compliance [8]. However, specific challenges in the dental setting remain, including long chair time, exposure to dental sounds (e.g., suction and drills) and physical proximity to the clinician, all of which contribute to dental fear [9]. While conventional VR systems may reduce anxiety to some extent, they lack continuous interaction and real-time responsiveness. The advantage of AI-enhanced VR systems is that they allow constant monitoring of the child's emotional state via facial recognition, pulse rate monitoring and eye-tracking technologies. These inputs enable the system to adjust content, such as altering visual scenes, audio tones, or introducing calming narratives whenever anxiety is detected [10]. Such personalization helps children remain focused and provides better emotional control. Additionally, AI can collect anonymized behavioral data to optimize future therapeutic environments, improving long-term outcomes. Behaviorally, AI-integrated VR systems also align with cognitive-behavioral principles of distraction, desensitization and positive reinforcement. The immersive, experience-based environment allows children to disengage from the clinical reality, while the AI-driven adaptability ensures relevance and calm in real time. Importantly, this approach reduces reliance on sedative or pharmacological interventions, making it safer and more accessible. Pediatric dentists and caregivers may find AI-driven VR systems especially useful for children with special healthcare needs or high levels of dental anxiety [11]. Even though the concept of AI-driven adaptive VR systems shows promise, there is limited research specifically examining their application in pediatric dentistry. Therefore, it is of interest to report the effectiveness of AI-integrated VR systems in minimizing dental anxiety in children compared with traditional behavioral management techniques.

## Materials and Methods:

A total of 60 children aged 6 to 12 years, requiring routine dental procedures such as oral prophylaxis or Class I restorations, were recruited and randomly allocated into two groups using a computer-generated randomization method. Group A (control group, n=30) received standard behavior management techniques, including tell-show-do and verbal reassurance. Group B (intervention group, n=30) was exposed to an AI-integrated Virtual Reality (AI-VR) environment 5 minutes prior to and during the dental procedure. The AI-VR system comprised a head-mounted display and headphones, connected to a biosensory unit capable of monitoring heart rate and facial expressions. The artificial intelligence algorithm was programmed to modify the VR content (e.g., changing scenes, background sounds, and voice tones) in real time based on emotional and physiological feedback. Calibration and standardization of the system were conducted before the intervention phase. Dental anxiety was assessed using the Modified Child Dental Anxiety Scale (MCDAS) both before and after the procedure. Additionally, physiological parameters such as heart rate and oxygen saturation were recorded using a pulse oximeter. Behavioral responses were evaluated using the Frankl Behavior Rating Scale by a blinded pediatric dentist. All data were compiled and analyzed using SPSS version 26.0. Independent t-tests and paired t-tests were applied for intra- and inter-group comparisons. A p-value of < 0.05 was considered statistically significant.

## Results:

The study involved 60 pediatric patients aged between 6 and 12 years, evenly distributed into two groups. Group A received conventional behavior management techniques, whereas Group B was exposed to an AI-integrated Virtual Reality system. Both groups were comparable in terms of age and gender distribution, with no statistically significant differences in baseline characteristics (Table 1 - see PDF). Baseline anxiety scores, recorded using the Modified Child Dental Anxiety Scale (MCDAS), showed no significant difference between groups prior to the intervention. However, post-treatment scores demonstrated a statistically significant reduction in anxiety in Group B (AI-VR) compared to Group A. Group B had a mean anxiety reduction of 9.3 points, whereas Group A showed a reduction of only 3.8 points (Table 2 - see PDF). Physiological responses also supported these findings. The average intraoperative heart rate was significantly lower in Group B, indicating better anxiety control. Additionally, behavioral assessment using the Frankl Behavior Rating Scale revealed more cooperative behavior in the VR group, with 80% of children scoring positive or definitely positive responses, as compared to 53.3% in the control group (Table 3 - see PDF). Overall, the AI-VR group exhibited significantly lower anxiety levels and more favorable physiological and behavioral outcomes (Tables 2 - see PDF and 3 - see PDF).

## Discussion:

Dental anxiety is one of the frequently occurring behavioral issues in children and may have great implication in providing quality dental treatment. The current study examined the effectiveness of an AI-linked Virtual Reality (VR) based intervention to lessen symptoms of anxiety in pediatric dental patients and showed marked enhancement in psychological and physiological perceptions between children, who received AI-VR intervention and those received customary methods of behavior management. The role of the immersive VR environment, as a distraction method that occupies the attention of the child away during the dental procedure has been constant and consistent in previous studies [1, 2]. Nevertheless, the conventional VR technologies cannot react to the individual emotional status in a real-time manner, and this might be a limitation of applying this technology to highly anxious children [3]. The thought-leading solution is possible to achieve through techniques combining artificial intelligence with VR and enabling real-time adjustment of the content according to the biometric and behavioral data related to the heart rate, facial expressions, eye movement, etc. [4, 5]. This reciprocity of feedback promotes the in-depth experience and keeps the child interested in the process, leading to a decreased level of anxiety and increased cooperation [6]. Through MCDADS, heart rate, and blood pressure, the AI-VR group, relative to the control group, has significantly decreased in our study. These results are consistent with previous studies where markers of physiological measures such as pulse rate and cortisol levels were lesser in children who had been exposed to immersive distractors [7, 8]. The virtual reality climate might be customized to every child with the help of AI which, most likely, helped achieve such high levels of anxiety reduction. The modulation of the scene and relaxing audio visual effects in real-time managed to keep the patient at a constant state of psychological stability throughout procedures likely to be distressing [9]. Moreover, the results of behavioral testing with Frankl Scale demonstrated more compliance on the part of the AI-VR group, which justified the conjecture about the improvement of assistance over the changes associated with lower traffic [10]. The use of AI in pediatric dentistry is an idea that is still quite new yet has a huge potential. Not only does it make live interaction possible, but it also makes it possible that the system becomes increasingly improved via machine learning and predictive modeling, using the amount of data recorded previously about the users [11]. This feature has the ability to boost personalization and effectiveness in the upcoming uses. Our results contribute to an emerging amount of data indicating that AI-based interventions can be superior to conventional behavioral strategies in managing anxiety in children [12]. Furthermore, the dissimilarity of AI-VR system is that it is non-invasive and non-pharmacologic, thus, is considered a favorable offer in children who have contraindications towards pharmacologic sedation [13]. Certain limitations should be noted even though it has shown a certain level of promise. The sample size was small and restricted to one institution, a factor that, to an extent, lowers the applicability of the results. Also, there was no follow-up after a long duration to determine the effects of the anxiety reducing effects during later visits to the dentist. The future work of research needs to include multicentric trials including greater sample size and must determine the long-term psychological benefits and the possibility of conditioning effect on behaviors [14]. The ability to employ additional elements of AI, like the natural language processing and adaptive storytelling to gain an even greater therapeutic potential of the system is also possible [15].

## Conclusion:

AI-integrated VR could be viewed as a promising instrument of promoting pediatric patients, helping them relax and feel less anxious about dental visits and behave better during these procedures is shown. The results point to the opportunity of joining immersive technology with intelligent systems to the management of behavior in children, bringing new aspects of patient-centered digital dentistry.
